# What Nurses Should Seek and Find

**Published:** 1918-03-16

**Authors:** 


### NURSING AFFAIRS IN IRELAND.

### WHAT NURSES SHOULD SEEK AND FIND.

A fatal error in warfare is to underrate the power of the enemy, and in the fierce struggle for victory between truth and falsehood now in progress in the nursing world this fact must not be forgotten. The turbulent and unscrupulous women who are the apostles of faction are not out for the benefit of the nurse, nor is their grievance concerned with democracy or freedom, or with any of the causes which concern the workers, any more than the commander of a pirate submarine is concerned with the freedom of the seas. They are a coterie, each one of whom is suffering from pique. Before the war they formed a mutual admiration society, talking much and accomplishing nothing, exploiting the nursing profession for their own aggrandisement, or for the gratification of their vanity. The war, however, has wrought a revolution, and has revealed to the civil and armed population alike what had never before been realised or appreciated—namely, that the trained nurse is a national asset of inestimable value. The last persons, probably, to grasp the truth of this are the nurses themselves. Their value to the sick and wounded is like wine that needs no bush. Hundreds of them have sacrificed their lives, and accomplished as much to glorify and sanctify their calling as Florence Nightingale did at the Crimea. It is of the highest importance that they should try to climb to the mountain-top and take a wide and comprehensive view of what has happened. They will then clearly see that the fanction-mongers who are sounding their little tin trumpets are part and parcel of the old *régime* swept aside by the revolution. They had an opportunity of rising with the flood, but they failed even to realise that there was a flood, and now they stand in danger of being submerged. Hence their pique, not to say their rage and their Bedlam. Listen to their insincere screeching about "charity" because the nation desires to honour the nursing profession! Have they forgotten that the trained nurse owes her very origin to the nation's tribute to Florence Nightingale? Have they forgotten that a "charity" offering of £30,000, subscribed by a grateful people after the long night of the Crimean campaign was the means of establishing British nurses in the solid position which they have since occupied? If they have, it may be no harm to remind them, though it will not affect the faction-mongers' attitude, for they are no more concerned with the question of charity than with that of democracy. These agitators are alone concerned because they realise that their day is nearly done, and their shibboleths are like straws at which drowning men clutch.

Nevertheless, the power which these women agitators possess must not be underrated. It is possible for them at the present moment effectually to prevent a Registration Bill for Nurses becoming law. The dames of the Irish Nurses' Association by themselves can without difficulty accomplish this feat, and I want the nursing profession to realise the truth. Half a dozen of them can gather together in a parlour in St. Stephen's Green, Dublin, prepare a *pot pourri* of Democracy spiced with Nationality, and send a dish to every Irish member of Parliament, with an S.O.S. cry for help. Nothing more is necessary. It is the easiest thing in the world, and they know it. They themselves have told me so. It matters not that nobody elects them: that nobody commissions them to act. They are there, out for mischief, ready to use any weapons that may help them to accomplish their wrecking mission, and just as unconcerned for the welfare of the working nurse as they were in their palmiest days.

Which brings me to my argument. The supreme consideration is not legislation, but union. A solid phalanx of British nurses can accomplish anything. They can move mountains, though the tin-trumpet brigade call their loudest. It is not their Registration Act that enables the medical profession to fight their battles, but unswerving devotion and loyalty to a common cause. Scratch a doctor, and you arouse an army. Just now the Irish dispensary doctors are threatening to "down tools" because they regard their salaries as inadequate. The expression is not mine, but theirs, and the Irish Press is full of the subject. By united action they will win, but not because of any Act of Parliament. My argument is a bread-and-butter argument. I do not seek to set it on any higher pedestal. It is an argument for to-day and for to-morrow. The trained nurse has the nation's ear, and it is the bounden duty of every working member of the nursing profession to see to it that she does not miss the tide. IERNE.

